# Raisins and additional walking have distinct effects on plasma lipids and inflammatory cytokines

**DOI:** 10.1186/1476-511X-7-14

**Published:** 2008-04-16

**Authors:** Michael J Puglisi, Ushma Vaishnav, Sudeep Shrestha, Moises Torres-Gonzalez, Richard J Wood, Jeff S Volek, Maria Luz Fernandez

**Affiliations:** 1 Department of Nutritional Sciences University of Connecticut, Storrs, CT 06269, USA; 2 Springfield College, Springfield, MA, 01109, USA; 3 Department of Kinesiology, University of Connecticut, Storrs, CT 06269, USA

## Abstract

**Background:**

Raisins are a significant source of dietary fiber and polyphenols, which may reduce cardiovascular disease (CVD) risk by affecting lipoprotein metabolism and inflammation. Walking represents a low intensity exercise intervention that may also reduce CVD risk. The purpose of this study was to determine the effects of consuming raisins, increasing steps walked, or a combination of these interventions on blood pressure, plasma lipids, glucose, insulin and inflammatory cytokines.

**Results:**

Thirty-four men and postmenopausal women were matched for weight and gender and randomly assigned to consume 1 cup raisins/d (RAISIN), increase the amount of steps walked/d (WALK) or a combination of both interventions (RAISINS + WALK). The subjects completed a 2 wk run-in period, followed by a 6 wk intervention. Systolic blood pressure was reduced for all subjects (P = 0.008). Plasma total cholesterol was decreased by 9.4% for all subjects (P < 0.005), which was explained by a 13.7% reduction in plasma LDL cholesterol (LDL-C) (P < 0.001). Plasma triglycerides (TG) concentrations were decreased by 19.5% for WALK (P < 0.05 for group effect). Plasma TNF-α was decreased from 3.5 ng/L to 2.1 ng/L for RAISIN (P < 0.025 for time and group × time effect). All subjects had a reduction in plasma sICAM-1 (P < 0.01).

**Conclusion:**

This research shows that simple lifestyle modifications such as adding raisins to the diet or increasing steps walked have distinct beneficial effects on CVD risk.

## Introduction

Cardiovascular disease (CVD) is the leading cause of death for both men and women in the United States [1]. Risk factors include elevated LDL cholesterol (LDL-C), triglycerides (TG), and blood pressure, low HDL cholesterol (HDL-C), and insulin resistance [2]. Implementation of effective diet and exercise interventions is necessary to reduce risk for CVD by improving these factors. Diet changes may include greater fruit intake; it has been estimated that increasing fruit intake by 1 serving/d reduces the risk for CVD by 7% [3]. Fruits may decrease the risk for CVD by providing dietary fiber, thus lowering plasma LDL-C [4], improving insulin sensitivity [5], and preventing atherosclerosis via their anti-inflammatory and antioxidant properties [6]. Regular exercise has been reported to aid in the prevention of CVD by decreasing TG and increasing HDL-C [7], increasing insulin sensitivity [8], and reducing oxidative stress [9].

The addition of raisins to the diet may decrease CVD risk, as they contain dietary fiber to lower LDL-C [10], as well as a significant amount of polyphenols [11]. Raisin polyphenols may interfere with cholesterol absorption, as shown with red wine polyphenols [12]. Raisins and red wine are both derived from grapes; however, the drying process causes loss of polyphenols in raisins [13]. Despite this, there is still a substantial amount of polyphenols on a per weight basis [13]. Raisin polyphenols can potentially decrease plasma TG by reducing apo E, as shown with lyophilized grape powder (LGP) supplementation in women [14]. LGP also decreased VLDL particle secretion from the liver [15], possibly via MTP inhibition [16,17], which would contribute to reduced plasma TG and LDL-C [6]. Polyphenols may act as antioxidants, further lowering CVD risk. Red wine polyphenols improved markers of oxidative stress in vitro [18,19] and reduced adhesion molecules in men [20]. Polyphenols also potentially reduce superoxide, decreasing its interaction with nitric oxide (NO) to improve vasorelaxation and blood pressure [19].

Regular exercise can decrease risk for CVD by decreasing TG and increasing HDL-C [7]. Exercise in the form of walking also has the potential to decrease LDL-C [21]. Exercise training may also reduce inflammation by decreasing TNF-α [22] and other inflammatory cytokines [23]. Moderate aerobic exercise decreased ICAM-1 in diabetic [24] and obese subjects [25] in previous research.

The purpose of this study was to determine if consumption of raisins, increasing steps walked, or a combination of these interventions affect risk for CVD by assessing resting blood pressure, plasma lipids, glucose, insulin and inflammatory cytokines.

## Results

As indicated in Table 1, there were no significant differences in age, body weight or blood pressure among groups of subjects at baseline.

**Table 1:** Characteristics of Subjects at Baseline^1^

	RAISIN (n = 12)	WALK (n = 12)	RAISIN + WALK (n = 10)
Age (yr)	54.4 ± 3.5	55.0 ± 3.8	57.8 ± 5.2
Weight (Kg)	70.8 ± 12.2	78.7 ± 16.8	78.6 ± 16.1
BMI (kg/m^2^)	24.9 ± 2.3	27.9 ± 3.9	27.5 ± 3.8
WC (cm)	86.4 ± 8.2	90.5 ± 13.4	91.0 ± 11.0
% Women	50% (6)	50% (6)	50% (5)
Diastolic blood pressure (mm Hg)	79.7 ± 10.6	78.0 ± 5.8	78.3 ± 15.9
Systolic Blood Pressure (mm Hg)	124.3 ± 15.2	120.7 ± 9.9	123.0 ± 14.0

^1 ^Values are expressed as mean ± SD for the number of subjects indicated in parentheses.

### Anthropometrics and Blood Pressure

Body weight and waist circumference were not changed as a result of the intervention (Table 2). In contrast, there was a significant 2.2% reduction for systolic pressure (P = 0.008) for all subjects after the intervention, regardless of group (Table 2). Diastolic blood pressure was not significantly altered.

**Table 2:** Anthropometrics and Blood Pressure. Body mass, systolic blood pressure (BP), diastolic blood pressure and waist circumference (WC) of subjects consuming raisins (RAISIN), increasing walking (WALK) or both (RAISIN + WALK)^1^.

**Variable**	**Body Mass (kg)**	**Systolic BP (mm of Hg)**	**Diastolic BP (mm of Hg)**	**WC (centimeters)**
**RAISIN (n = 12)**				
Baseline	70.8 ± 12.2	124.3 ± 15.2	79.7 ± 10.6	86.4 ± 8.2
Week 6	70.9 ± 11.9	121.8 ± 13.7	79.2 ± 9.1	85.7 ± 9.1
**WALK (n = 12)**				
Baseline	78.7 ± 16.8	120.7 ± 9.9	78.0 ± 5.8	90.5 ± 13.4
Week 6	78.6 ± 17.1	118.7 ± 8.9	77.8 ± 5.2	90.5 ± 13.2
**RAISIN + WALK (N = 10)**				
Baseline	78.4 ± 15.9	123.0 ± 14.0	80.6 ± 8.4	91.0 ± 11.0
6 Weeks	78.4 ± 16.0	119.4 ± 13.3	78.8 ± 9.5	90.6 ± 11.8
Time Effect	NS	P = 0.008	NS	NS
Group Effect	NS	NS	NS	NS
**Interaction**	NS	NS	NS	NS

^1 ^Values represent mean ± SD for the number of subjects indicated in parentheses. Data were analyzed using repeated measures ANOVA. There was a time effect for blood pressure only.

### Dietary Analysis

Carbohydrate intake during the intervention was significantly higher for RAISIN (56.2% of total kcals) and RAISIN + WALK (57.1% of total kcals) than WALK (43.4% of total kcals, Table 3). Percentage of energy from dietary total fat, monounsaturated fat and polyunsaturated fat were significantly greater for WALK than RAISIN and RAISIN + WALK (Table 3). Dietary fiber was significantly greater for RAISIN and RAISIN + WALK than WALK (Table 3).

**Table 3:** Dietary Analysis. Percent (%) of energy from total fat, saturated (SAT), monounsaturated (MONO) and polyunsaturated (PUFA) fat, dietary cholesterol and dietary fiber during the intervention of subjects consuming raisins (RAISIN), increasing walking (WALK) or both (RAISIN + WALK)^1^.

	**RAISIN (N = 12)**	**WALK (N = 12)**	**RAISIN + WALK (N = 10)**
Carbohydrate (%en)	56.2 ± 6.5^a^	43.4 ± 7.6^b^	57.1 ± 6.7^a^
Protein (%en)	15.1 ± 2.4	16.1 ± 4.5	15.3 ± 2.6
Total fat (%en)	30.8 ± 4.9^a^	39.6 ± 6.6^b^	29.1 ± 4.5^a^
SAT (%en)	9.5 ± 5.4	11.8 ± 3.0	8.9 ± 2.0
MONO (%en)	11.0 ± 1.7^a^	14.7 ± 3.2^b^	10.6 ± 2.3^a^
PUFA (%en)	6.2 ± 2.6^a^	8.9 ± 2.7^b^	6.3 ± 2.2^a^
Fiber (g)	25.7 ± 5.1^a^	19.1 ± 6.6^b^	27.1 ± 6.9^a^
Cholesterol (mg)	231.8 ± 106.8	282.8 ± 89.9	319.6 ± 126.1

^1 ^Values represent mean ± SD for the number of subjects indicated in parentheses. Values in the same row with different superscripts are significantly different as determined by one-way-ANOVA and the least significance test.

### Plasma Lipids

There was a significant time effect for plasma total cholesterol (P < 0.005, Table 4), as concentrations were reduced by 9.4% from baseline to post-intervention. However, there was no group effect on plasma total cholesterol. The decrease in plasma total cholesterol can be explained mainly by the 13.7% reduction in plasma LDL-C (P < 0.001, Table 4). Plasma HDL-C was unchanged for the subjects as a result of the intervention. There was a significant group effect for plasma TG (P < 0.05), as concentrations were unchanged for RAISIN and RAISIN + WALK (Table 4), but plasma TG was reduced by 19.5% for WALK (Table 4).

**Table 4:** Plasma Lipids. Plasma total cholesterol (TC), LDL cholesterol (LDL-C), HDL cholesterol (HDL-C) and triglycerides (TG) of subjects consuming raisins (RAISIN), increasing walking (WALK) or both (RAISIN + WALK)^1^.

Variable	TC (mmol/L)	LDL-C (mmol/L)	HDL-C (mmol/L)	TG (mmol/L)
**RAISIN (n = 12)**				
Baseline	5.21 ± 0.98	3.21± 0.84	1.56 ± 0.36	0.94 ± 0.47
6 Weeks	4.82 ± 0.93	2.90 ± 0.76	1.53 ± 0.31	0.90 ± 0.35
**WALK (n = 12)**				
Baseline	5.47 ± 1.37	3.16 ± 1.16	1.56 ± 0.50	1.49 ± 0.99
6 Weeks	4.68 ± 1.12	2.48 ± 0.60	1.58 ± 0.41	1.20 ± 0.73
**RAISIN + WALK (n = 10)**				
Baseline	5.25 ± 0.96	3.01 ± 1.11	1.71 ± 0.50	1.17 ± 0.53
6 Weeks	4.96 ± 0.79	2.74 ± 0.71	1.66 ± 0.55	1.24 ± 1.54
Time Effect	P < 0.005	P < 0.001	NS	NS
Group Effect	NS	NS	NS	P < 0.05
Interaction	NS	NS	NS	NS

^1^Values are means ± standard deviation for the number of subjects indicated in parenthesis. Data were analyzed using repeated measures ANOVA.

### Steps per day, Plasma Insulin and Glucose

Steps taken/d, as estimated by pedometers, were significantly increased during the intervention for WALK and RAISIN + WALK (P < 0.0001, Table 5). Plasma insulin and glucose were unchanged as a result of the interventions (Table 5).

**Table 5:** Steps Walked, Glucose and Insulin. Number of steps, plasma glucose and insulin of subjects consuming raisins (RAISIN), increasing walking (WALK) or both (RAISIN + WALK)^1^.

Variable	Number of Steps	Glucose (mmol/L)	Insulin (mmol/L)
**RAISIN (N = 12)**			
Baseline	N/A	5.52 ± 0.71	402 ± 278
6 Weeks	N/A	5.38 ± 0.68	391 ± 326
**WALK (n = 12)**			
Baseline	6083 ± 2639	5.52 ± 0.70	317 ± 157
6 Weeks	11998 ± 4022	5.70 ± 0.93	315 ± 180
**RAISIN + WALK (n = 10)**			
Baseline	9481 ± 3641	5.22 ± 0.41	316 ± 134
6 Weeks	12547 ± 4329	5.40 ± 0.54	349 ± 205
Time Effect	P < 0.0001	NS	NS
Group Effect	NS	NS	NS
Interaction	NS	NS	NS

^1^Values are means ± standard deviation for the number of subjects indicated in parenthesis. Data were analyzed using repeated measures ANOVA. There were no interactive effects for these variables.

### Plasma Inflammatory Cytokines

**Figure 1:**
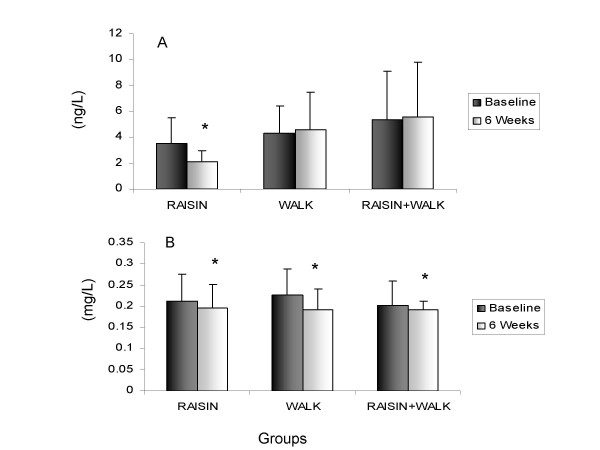
**Changes in TNF-α (Panel A) and sICAM (Panel B) between baseline and 6 wk for subjects in RAISIN (N = 12), WALK (n = 12) or RAISIN + WALK (n = 10 groups).** * indicates significantly different from baseline (P < 0.025). For TNF-α, there was a time effect only for the raisin group as determined by Tukey post-hoc test.

Plasma IL-8 and MCP-1 did not change for the intervention, although there was a trend for a decrease in raisins for MCP-1 (P = 0.078). In contrast, there was a significant group and group × time effect on plasma TNF-α concentrations (Fig 1, panel A); as values were not changed for WALK or RAISIN + WALK, but were significantly decreased from 3.5 ng/L to 2.1 ng/L from baseline to post-intervention for RAISIN (P < 0.025). There was a significant time effect for plasma sICAM-1 (P < 0.01); concentrations were significantly decreased for all groups, with no differences between groups (Fig. 1, Panel B).

## Discussion

This study showed that raisins, walking, or a combination of these interventions have distinct beneficial effects on risk for CVD. Plasma lipids were significantly affected for all groups, with significant reductions in total cholesterol and LDL-C. The intervention resulted in a reduction in LDL-C from 3.13 mmol/L to 2.71 mmol/L, which is clinically significant, bringing the subjects close to the optimal target, below 2.60 mmol/L. The increase in fiber intake was a likely contributor to the reduction in LDL-C for RAISIN and RAISIN + WALK. The increase in dietary fiber interferes with enterohepatic circulation of bile, increasing bile acid excretion [26]. The depletion of hepatic cholesterol then results from an increase in bile acid synthesis to replace losses, with a subsequent increase in expression of the hepatic LDL receptor to reduce plasma LDL-C [4].

Polyphenols provided by raisins may interfere with cholesterol absorption [6], decreasing hepatic cholesterol concentrations, as reported with guinea pigs after supplementation with lyophilized grape powder (LGP) [15]. As a result of lower hepatic cholesterol concentrations, hepatic LDL receptor expression increases to enhance cholesterol uptake from LDL, thus lowering plasma LDL-C. A reduction in plasma LDL-C was also reported with LGP supplementation in women [14]. Naringenin, a polyphenol found in grapefruit, has been shown to inhibit microsomal transport protein (MTP) [16,17]. This could be another potential mechanism through which LDL-C is lowered with raisin intake. If MTP is inhibited, less lipid is transferred to apo B, increasing the susceptibility of apo B to degradation [17]. As a result, VLDL secretion is decreased, with a subsequent lowering of LDL-C.

Exercise may increase LPL [27], which can lead to quick removal of VLDL particles from the circulation, decreasing LDL-C. If LDL particles are rich in TG, apoB 100 receptor binding can be altered, reducing uptake by LDL receptor [28]. Thus, the reduction in TG for WALK (explained below) may have enhanced LDL uptake, decreasing LDL-C. The quick removal of triglyceride-rich lipoproteins may also reduce the potential for cholesteryl ester transfer protein (CETP) to act on these particles [29]. This decreases the transfer of cholesteryl esters from HDL particles to triglyceride-rich lipoproteins, including LDL and VLDL particles (which are degraded to LDL particles); lowering plasma LDL-C.

The reduction in TG was expected with initiation of exercise, via 2 possible mechanisms: 1) an increase in TG removal with elevated LPL, or 2) decreased TG secretion from the liver [27]. Exercise activates AMP-activated protein kinase (AMPK) activity to potentially lower hepatic TG content and secretion of TG from the liver [30]. AMPK inhibits acetyl CoA carboxylase (ACC), the rate-limiting enzyme in fatty acid synthesis [31]. The decrease in malonyl CoA also lowers inhibition of carnitine palmitoyl transferase-1 (CPT-1), increasing fatty acid oxidation [31].

A synthetic polyphenol, as well as resveratrol, stimulated AMPK in vitro; leading to a reduction in TG accumulation [31]. The synthetic polyphenol also increased AMPK activity and decreased serum TG in LDL receptor-deficient mice [31]. However, the mice were given a very large dose (130 mg/kg/d) of the polyphenol [31]. It is questionable if raisin polyphenols worked through this mechanism in this study, given the lack of human evidence. LGP supplementation with humans caused a reduction in plasma TG [14], possibly by decreasing apo E concentrations, which increases LPL activity because there is less replacement of the LPL activator apo CII on the VLDL particle. In the current study there was no change in TG for RAISIN and RAISIN + WALK. This may have been a result of a decrease in plasma TG by polyphenols being offset by the increase in TG synthesis and secretion as a result of increased carbohydrate intake [27].

The reduction in blood pressure for RAISIN and RAISIN + WALK may have resulted from antioxidant effects of the raisin polyphenols. Scavenging of ROS inhibits NAD(P)H oxidase and subsequently decreasing superoxide production [19]. This lowers nitric oxide (NO) degradation by preventing interaction of superoxide with NO to form the highly reactive radical peroxynitrite [19]. A decrease in superoxide may also lower transcription of the redox-sensitive transcription factor activator protein (AP)-1 [32]. This would reduce transcription of the vasoconstrictor endothelin-1 (ET-1). The end result is vasorelaxation, and improved blood pressure, as well as improved endothelial dysfunction [19], which is important since endothelial dysfunction increases risk for atherosclerosis by promoting vasoconstriction, platelet aggregation, thrombosis, and adhesion of monocytes to the endothelium [33]. Walking may have decreased blood pressure by causing vascular remodeling, altering sympathetic nerve activity and decreasing the sensitivity of the blood vessels to ET-1 [34]. The clinical significance of the change in blood pressure may be questioned; a change in systolic blood pressure of 2.6 mm Hg may not be meaningful. It is likely that the small change resulted from the fact that the subjects were normotensive to start the study, as normotensive individuals typically do not have as large of a change in blood pressure with treatment as hypertensive subjects [34].

The lack of change in plasma glucose and insulin for RAISIN and RAISIN + WALK is expected given that plasma TG were unaffected by the interventions. A decrease in TG may lower insulin resistance by alleviating lipotoxicity [31]. The increase in fat oxidation and reduction in fat breakdown in the tissues aids in the decrease in lipid accumulation, preventing interference with insulin receptor signaling and improving insulin sensitivity [31]. Based on this, an improvement in plasma glucose and insulin may be expected for WALK. However, the fact that these subjects were insulin-sensitive prior the intervention may have contributed to the nonsignificant changes in these variables.

Raisins and walking had positive effects on markers of inflammation. It is expected that the inflammatory cytokines would be significantly decreased for RAISIN and RAISIN + WALK, as polyphenols may act to lower oxidative stress and inflammation [6,19,20]. Green tea extract was found to reduce the gene expression of TNF-α as well as decrease its protein concentration in the lungs of mice [35]. Although they are present in much smaller amounts, various catechins in green tea can also be found in raisins [13]. This may contribute to the decrease in TNF-α for RAISIN.

Oral gavage of large doses of resveratrol significantly decreased TNF-α concentrations in rats [36]. However, moderate consumption of red wine failed to affect TNF-α concentrations in previous research with healthy subjects similar in age to the subjects in this study [20,37]. The inconsistency in other studies appeared to be present in this experiment as well, as TNF-α was not significantly altered for RAISIN + WALK. More research is necessary to determine if raisin polyphenols decrease TNF-α expression, potentially inhibiting NFκB transcription of inflammatory cytokines and chemoattractant and adhesion molecules that play a key role in progression of atherosclerosis.

Researchers have indicated that regular aerobic exercise inhibits the production of TNF-α, potentially via an increase in IL-6 with each acute bout [38]. IL-6 has been shown to promote anti-inflammatory cytokines and inhibit TNF-α [39-41]. It is probable that the acute walking bouts in this study affected IL-6 [42]. However, exercise intensity and duration, as well as skeletal muscle mass recruitment are important factors in IL-6 elevation with exercise [43], possibly limiting the effects of IL-6 on TNF-α in this study. TNF-α is sometimes decreased with exercise training, but this alteration is not consistent [44,45], and a long intervention may be necessary [46]. The short duration of this study as well as the mild nature of the exercise may have contributed to the lack of alteration in plasma TNF-α for WALK and RAISIN + WALK.

The decrease in sICAM-1 for RAISIN and RAISIN + WALK is supported by findings from previous research by Estruch et al. [20], where ~2 glasses of red wine/d decreased ICAM-1 in healthy men. Coimbra et al. [47] also reported a decrease in ICAM-1 with consumption of grape juice. This reduction may be a result of a reduction in NFκB activation, which would decrease the expression of ICAM-1, as well as other inflammatory markers and adhesion molecules [48]. TNF-α increases expression of ICAM-1 via activation of NFκB [49,50]. Therefore, it is possible that the reduction in TNF-α for RAISIN contributed to the decrease in sICAM-1. The decrease in ICAM-1 for WALK is in agreement with studies involving moderate exercise training by Zoppini et al. [24] and Rector et al. [25]. The cause of the decrease in ICAM-1 may have been different since TNF-α was not altered by exercise. Oxidants such as hydrogen peroxide have been shown to affect ICAM-1 expression by activating the transcription factors AP-1 and Ets [51]. An improvement in antioxidant status with exercise would contribute to the lowering of sICAM-1 for WALK.

In conclusion, risk factors for CVD were affected significantly by consuming raisins or increasing steps walked/d. Blood pressure, plasma total cholesterol and LDL-C were significantly decreased by all interventions, while walking lowered plasma TG. All interventions significantly reduced plasma concentrations of sICAM-1, potentially preventing progression of atherosclerosis by decreasing adhesion of monocytes to the vascular endothelium. Raisins lowered the risk for inflammatory damage by decreasing TNF-α. The findings of this research show that simple lifestyle modifications such as adding raisins to the diet or increasing steps walked/d have distinct beneficial effects on risk factors for CVD.

## Methods

### Materials

Kits for total cholesterol (TC) and triglycerides were from Roche Diagnostics (Indianapolis, IN); glucose kits were from WACO (Waco Diagnostics, Richmond, VA).

Raisins were provided by the California Table Grape Commission (Fresno, CA).

### Subjects

Men and postmenopausal women between the ages of 50–70 y were recruited from the University community for this study. Subjects provided informed consent and completed a medical history during recruitment. Exclusion criteria included taking blood-thinning medications, cigarette smoking, diabetes, cardiovascular disease, or renal disease. Subjects with a body mass index greater than 37 kg/m^2 ^were also excluded from the study. A total of 17 men and 17 postmenopausal women volunteered to participate in the study. Study protocols were approved by the Institutional Review Board.

### Study Design and Randomization

The subjects in the study were matched according to sex and body mass, then randomly assigned to 1 of 3 groups: 1) a group that consumed 1 cup raisins/d (RAISIN), 2) a group that increased the amount of steps taken each day (WALK), or 3) a group that consumed 1 cup raisins/d and increased the amount of steps taken (RAISIN + WALK). The subjects completed a 2 wk washout period, followed by a 6 wk intervention.

### Diet and Exercise Description

All subjects completed a 2 wk run-in period in order to standardize exercise and dietary habits. Subjects were asked to maintain there normal level of activity and abstain from polyphenol-rich foods, including grapes, berries, wine, chocolate, raisins, tea, vitamins, and any other supplements. These restrictions were continued during the 6 wk intervention in order to isolate the antioxidant effects of the raisins. The subjects that were asked to increase the amount of steps walked/d during the intervention were issued a pedometer. The subjects maintained their normal daily activity during the 2 wk period in order to obtain an estimate of the amount of steps/d. The subjects in RAISIN were asked to maintain their normal level of activity during the 6 wk intervention.

The raisins consumed by the subjects in this study were provided by the researchers weekly with a checklist to determine compliance. A Registered Dietitian provided dietary instruction for substituting the raisins for other foods to ensure weight maintenance, and provided written materials to reinforce the information. The dietitian also counseled WALK subjects to ensure weight maintenance during the intervention. The subjects were encouraged to consume the raisins with other foods and space their intake throughout the day in order to make consumption easier. Subjects were also instructed on how to accurately complete a food frequency questionnaire and a detailed food record.

WALK participants were instructed to increase their steps by walking an additional 10 min/d (above their normal activity) every 2 wk in an attempt to increase their walking by approximately 1 km/d every 2 wk. Therefore, the subjects were walking an additional 10 min/d for the first 2 wk, 20 min/d for the second 2 wk, and 30 min/d for the last 2 wk (or the equivalent of an additional 3 km/d). The subjects logged their steps daily, and noted any difficulties with the pedometer or unusual physical activity (i.e., less walking due to illness, inclement weather or traveling all day).

### Data Collection

Subjects completed a food frequency questionnaire after random assignment into groups to assess usual dietary intake. A physical activity questionnaire was also administered to estimate average activity levels. After the baseline information was collected, the subjects reported to the laboratory following an overnight fast (~12 h) at the beginning of the 2 wk washout period. At this point, pedometers were issued to subjects in the WALK and RAISIN + WALK groups, and subjects began the dietary restrictions. A phlebotomist collected 10 mL of blood from the antecubital vein into a tube containing EDTA to assess lipids for screening purposes.

The subjects returned to the laboratory twice more at the start of the 6 wk intervention after the 2 wk washout period. Blood was drawn as before; 60 mL in the first visit, and 10 mL in the second visit (just for assessment of lipids). Body weight, height, waist circumference and resting blood pressure were also measured for each subject in the first visit. Plasma was separated by centrifugation at 2200 × g for 20 min at 4°C; and sodium azide (1 μL/mL), phenylmethyl-sulphonyl fluoride (PMSF; 1 μL/mL), and aprotinin (5 μL/mL) were added to the samples for preservation. Approximately 1 mL of plasma was aliquoted at both time points to assess plasma lipids. The remainder of the plasma was aliquoted into microcentrifuge tubes and stored at -80°C for later analysis. This procedure was completed again at the end of the 6 wk, as the subjects reported twice for blood collection in the same wk.

Subjects completed a 5 d diet record, including 3 weekdays and two weekend days, during the study to assess normal dietary consumption. The days were not necessarily consecutive, but it was emphasized that the days should represent the subjects' typical intake. Subjects in the RAISIN and RAISIN + WALK groups received sheets to record raisin consumption; and subjects in the WALK and RAISIN + WALK groups received forms to record daily step totals. These forms were distributed weekly, and a researcher reviewed the data with each subject weekly. Raisins were also distributed weekly. Counseling was provided at this time to ensure that subjects adhered to dietary restrictions and increased their steps as appropriate.

### Dietary Analysis

All dietary records were analyzed using the Nutrition Data System 5.0 (Minneapolis, MN). Total kcal intake, as well as carbohydrate, fat, protein, and dietary fiber were determined. Also, monounsaturated, polyunsaturated, and saturated fat was assessed, as well as dietary cholesterol and dietary fiber.

### Plasma Lipids

Plasma lipids were determined by taking the average of 2 values obtained on separate days of the same wk before and after the intervention. This was done to account for day-to-day variability in these values. Plasma total cholesterol was measured in triplicate using enzymatic methods, with Roche Diagnostics standards and kits [52]. Plasma TG was measured in triplicate utilizing Roche Diagnostics kits that adjust for free glycerol [53]. HDL-C was measured by precipitating apo B containing lipoproteins using magnesium chloride and dextran sulfate [54], then assessing the supernatant in triplicate with standards and kits from Roche Diagnostics. LDL-C was estimated by the Friedewald equation [55].

### Blood Pressure

Seated resting systolic and diastolic blood pressure were measured after 5 min of rest using a Welch Allyn Tycos blood pressure cuff.

### Plasma Glucose, Insulin and Cytokines

Plasma glucose was analyzed by an automated lactate/glucose analyzer (2300 STAT, YSI, Yellow Springs, OH). Plasma insulin and cytokines (TNFα, IL-8, MCP-1, tPAI-1, sICAM-1, and sE-Selectin) were measured using xMAP^® ^technology on a Luminex ^® ^IS 200 system with antibodies to these analytes from LINCO Research (St. Charles, MO) [56]. Assays were completed according to manufacturer's instructions.

### Statistical Analyses

All statistical analyses were performed with SPSS 12.0 for Windows (SPSS Inc., Chicago, IL). One way ANOVA was used to determine difference in macronutrient intake for the groups. The least significant difference was used as a post-hoc test. Repeated measures ANOVA was utilized to determine changes in variables over time for the three groups. If a significant interactive effect was found, Tukey post hoc analyses were completed. Significance was set at P ≤ 0.05.

## Competing interests

Supported by a grant from The California Grape Commission to MLF

## Authors' contributions

MLF designed the study, with suggestions and help from MP and JV. MP, UV, SS, MTG, and RW participated in carrying out the study and data collection. MP, UV, SS, and RW completed the assays. MP and MLF drafted the manuscript. All authors read and made suggestions for the final manuscript.
